# Elevated Extracellular cGMP Produced after Exposure to Enterotoxigenic Escherichia coli Heat-Stable Toxin Induces Epithelial IL-33 Release and Alters Intestinal Immunity

**DOI:** 10.1128/IAI.00707-20

**Published:** 2021-03-17

**Authors:** Natalya I. Motyka, Sydney R. Stewart, Ian E. Hollifield, Thomas R. Kyllo, Joshua A. Mansfield, Elizabeth B. Norton, John D. Clements, Jacob P. Bitoun

**Affiliations:** a Department of Microbiology and Immunology, Tulane University School of Medicine, New Orleans, Louisiana, USA; New York University School of Medicine

**Keywords:** enterotoxins, inflammation, intestinal immunity

## Abstract

Enterotoxigenic Escherichia coli (ETEC) is a major diarrheal pathogen in children in low- to middle-income countries. Previous studies identified heat-stable enterotoxin (ST)-producing ETEC as a prevalent diarrheal pathogen in children younger than 5 years. While many studies have evaluated the interaction of ETEC heat-labile enterotoxin (LT) with host epithelium and immunity, few investigations have attempted similar studies with ST. To further understand ST pathogenesis, we examined the impact of ST on cGMP localization, epithelial cell cytokine production, and antibody development following immunization. In addition to robust intracellular cGMP in T84 cells in the presence of phosphodiesterase inhibitors (PDEis) that prevent the breakdown of cyclic nucleotides, we found that prolonged ST intoxication induced extracellular cGMP accumulation in the presence or absence of PDEis. Further, ST intoxication induced luminal cGMP *in vivo* in mice, suggesting that secreted cGMP may have other cellular functions. Using transcriptome sequencing (RNA-seq) and quantitative PCR (qPCR), we demonstrated that ST intoxication, or treatment with the clinically used ST mimic linaclotide, altered inflammatory cytokine gene expression, including the interleukin 1 (IL-1) family member IL-33, which could also be induced by cell-permeative 8-Br-cGMP. Finally, when present during immunization, ST suppressed induction of antibodies to specific antigens. In conclusion, our studies indicate that ST modulates epithelial cell physiology and the interplay between the epithelial and immune compartments.

## Abstract (precis)

Enterotoxigenic Escherichia coli (ETEC) is a major diarrheal pathogen in children in low- to middle-income countries. Previous studies identified heat-stable enterotoxin (ST)-producing ETEC as a prevalent diarrheal pathogen in children younger than 5 years.

## INTRODUCTION

Enterotoxigenic Escherichia coli (ETEC) is a significant global cause of secretory diarrhea against which there are currently no licensed vaccines. Most ETEC cases occur in children younger than 5 years in low- and middle-income countries (LMICs) and in travelers and military personnel deployed to LMICs and other regions where ETEC is endemic (1). ETEC isolates are classified by their enterotoxin expression profiles based on the types and combinations of enterotoxins they produce: heat-labile enterotoxin-producing (LT^+^) ETEC, heat-stable enterotoxin-producing (ST^+^; human [STh] or porcine [STp]) ETEC, and LT^+^ ST^+^ ETEC. ETEC producing any combination of these toxins can cause secretory diarrhea in humans (2).

LT is a highly immunogenic protein able to induce antibodies following natural LT^+^-ETEC infection. LT binds to GM1 gangliosides on intestinal epithelial or immune cell surfaces and is internalized to modulate cyclic AMP (cAMP) signaling pathways. Numerous studies have focused on LT-mediated modulation of the host epithelium and immune system. LT induces expression of interleukin 1 (IL-1) family members, including IL-1β from murine jejunum and ileum (3) and IL-1α and IL-1β from murine dendritic cells (4), and potent T helper cell type 17 (T_H_17) and mucosal IgA responses (5, 6). In comparison, ST-specific pathogenesis remains relatively understudied despite the majority of the moderate to severe diarrheal cases resulting from ST^+^-ETEC or ST^+^ LT^+^-ETEC infection (2). ST is a small secreted peptide that fails to induce robust antibodies during natural ST^+^-ETEC exposure. ST competes with the endogenous peptides guanylin and uroguanylin for activation of the particulate guanylyl cyclase C (GC-C) receptor to induce cGMP production. ST-induced cGMP levels result in cGMP-dependent kinase C type II (cGKII) and, as with LT, protein kinase A (PKA) phosphorylation to activate CFTR (cystic fibrosis transmembrane conductance regulator) and inhibit NHE3 (sodium-hydrogen exchanger 3), resulting in secretory diarrhea (7). In fact, the ST analog linaclotide is used clinically to treat irritable bowel syndrome with symptoms of constipation (8). Activating mutations in GC-C in cases of familial *GUCY2C* diarrhea syndrome (FGDS) (9) as well as decreased expression of GC-C in ulcerative colitis (UC) (10) suggests that precise GC-C signaling is important to maintain intestinal health and homeostasis.

ST applied in a continuous flow to flexible jejunal monolayers induces cGMP in secreted but not intracellular fractions, suggesting the emergence of a role for secreted cGMP in ETEC pathogenesis. Additionally, ST induced both apical and basolateral secretion of cGMP in polarized enteroid monolayers, suggesting that ST intoxication could affect interactions between the epithelial and immune compartments via cGMP (11). In fact, small cyclic nucleotides have shown evidence of immune modulation: intranasal administration of house dust mite (HDM) and cyclic GMP-AMP (cGAMP) can promote T_H_2 responses (12). Moreover, it has been shown that ST reprograms epithelial signaling and that the inflammatory cytokines induced by LT in animals are damped in the presence of ST (3), interactions that could alter mucosal immune system function. Recent antibody analysis of sera from individuals challenged with ST-only ETEC TW10722 suggests that mucosal immune responses are induced in the near term, but the longevity of immune responses wanes over time (13). Also, during heterologous ETEC challenge studies, the majority of volunteers who developed anti-LT serological responses following LT^+^ ST^+^-ETEC B7A exposure were still susceptible to LT^+^-ETEC E2528-C1-mediated challenge (14), supporting the idea that ST hinders development of sustained anti-LT immune or anti-ETEC responses or that anti-LT responses alone are not sufficient for long-term protection.

Enteric pathogens like ETEC are transmitted via the fecal-oral route, and the development of mucosal immune responses involves many different cell types, cytokines, and receptors. Differentiation between homeostatic epithelial shedding and pathogen-mediated epithelial remodeling, including cytoskeletal F-actin polarization, is required to balance T_H_1 and T_H_2 immune responses (15). Because ETEC is typically noninvasive and adheres to host epithelium through structurally diverse adhesins called colonization factors (15), mucosal antibody responses are thought to mediate some type of protection from ETEC infection. In fact, oral delivery of hyperimmune bovine colostrum containing antibodies to CS17 fimbriae protects against ETEC-mediated diarrhea in human challenge studies (16).

However, productive immune responses following ETEC infection (and reinfection) are not typically induced until around the age of 5 years, which is surprising since LT variants boost the immune response to coadministered antigens (5, 6). Detoxified double mutant LT (dmLT; LT R192G/L211A) is a potent mucosal adjuvant (5) that boosts the immune response to orally delivered ETEC antigens in adults and 6-month-old infants (17). With promising ETEC clinical vaccine formulations in development, induction of anti-ETEC immunity may not be difficult. However, ST produced by ETEC may suppress the development of long-lasting ETEC immunity, whether induced by immunization or natural exposure, especially since most of the current ETEC vaccine candidates do not contain an ST antigen component. Thus, it is important to evaluate the role of ST during the induction of immune responses.

One knowledge gap regarding ETEC pathogenesis is understanding why some ETEC isolates expend the genetic and metabolic energy required to maintain and produce both ST and LT enterotoxins, given that each toxin causes secretory diarrhea via similar cyclic nucleotide-driven mechanisms. The previous rationale for secretory toxins is that they aide further dissemination of pathogens. However, we believe that ST has multiple yet-to-be defined roles in ETEC pathogenesis. Here, we wanted to understand the impact of ST and ST-induced cGMP on the interplay between the host intestinal epithelium and immune responses. The primary goal of this study was to determine if and how ST influences epithelial cell development and innate and adaptive immunity. The GC-C signaling cascade should also be better understood to inform therapeutic and prophylactic strategies to treat ST^+^ ETEC infection. Deepening our understanding of ST-specific pathogenesis may help to uncover the reason for suppression of neutralizing anti-ETEC immune responses in children in LMICs and why ST^+^ ETEC causes such severe cases of diarrhea in young children.

## RESULTS

### ST intoxication induces secreted cGMP.

**FIG 1:**
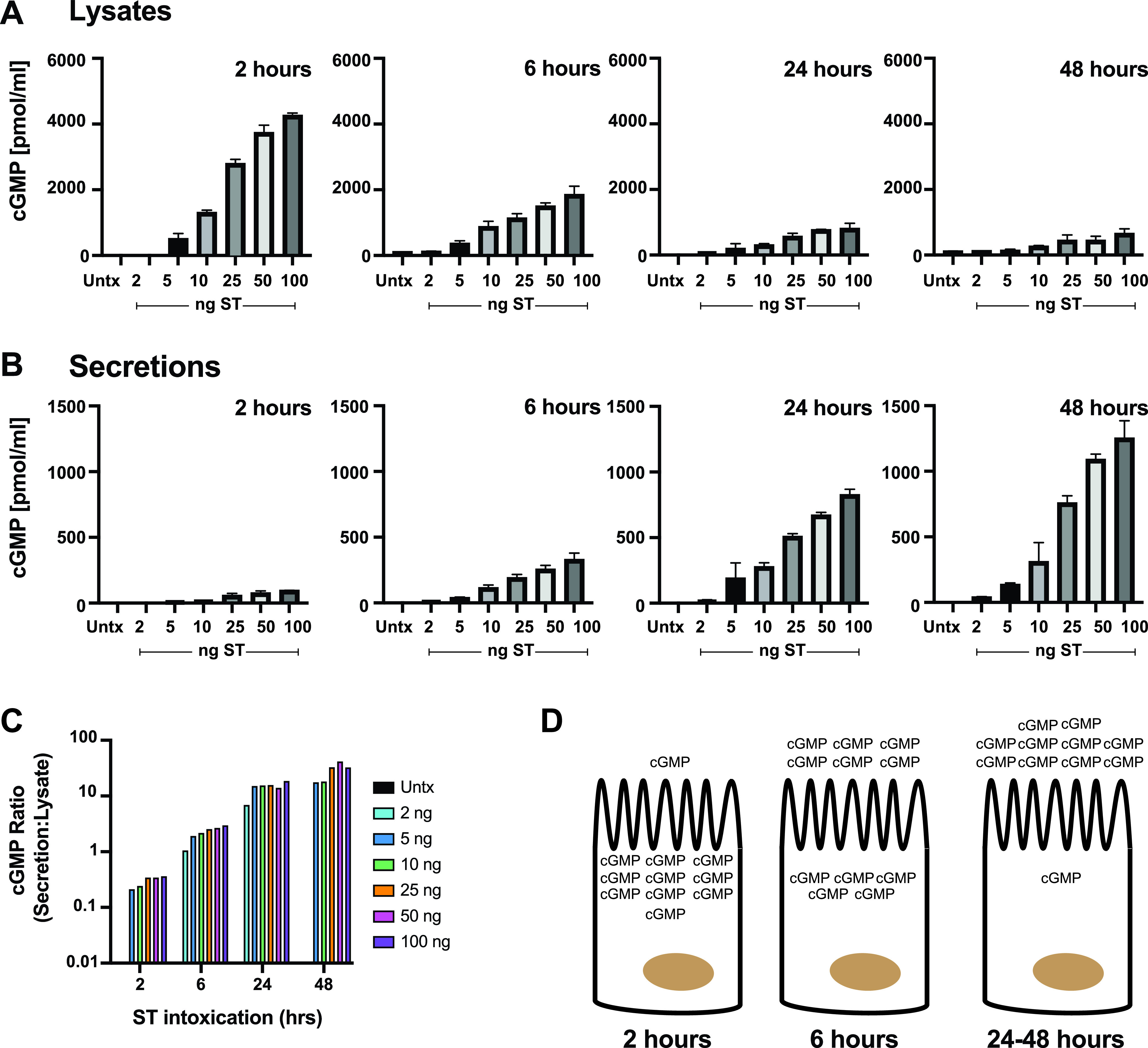
ST intoxication induces secreted cGMP in T84 epithelia. Saline (Untx) or ST (2, 5, 10, 25, 50, and 100 ng) was applied to T84 cells pretreated for 1 h with the phosphodiesterase inhibitors (PDEis) zardaverine (20 μM) and vardenafil (30 μM). cGMP was measured using a cGMP Parameter assay kit by R&D according to the manufacturer’s instructions in T84 lysates (A) and cell-free secretions (B) after 2, 6, 24, and 48 h of ST intoxication. The ratio of cGMP found in respective cell-free secretions to that in T84 cell lysates (C) shows that ST intoxication initially induced cGMP accumulation intracellularly (*t* = 2 h; the ratio of cGMP in secretions to lysates is less than 1), and then the ratio of cGMP in secretions to lysates reaches an equilibrium (*t* = 6 h; the ratio of cGMP in secretions to that in lysates is ∼1.0) before the majority of ST-induced cGMP was found extracellularly (*t* = 24 and 48 h; the ratio of cGMP in secretions to lysates is greater than 1.0). The kinetics of cGMP induction and secretion are illustrated in panel D. Data are averages from three independent experiments with three technical replicates per condition.

Recent investigations have shown that cGMP induced by ST intoxication can be found in cell-free supernatants of enteroid monolayers (11) at low levels, suggesting that cGMP may alert proximal cells of ETEC infection via autocrine or paracrine signaling. To further our understanding of the role of cGMP during ETEC infection, we first wanted to understand longevity of the cGMP induced by ST intoxication. Confluent T84 intestinal epithelial cell monolayers were pretreated with the phosphodiesterase inhibitors (PDEis) zardaverine and vardenafil to prevent the degradation of cGMP for 1 h before ST intoxication over a dose range (0, 2, 5, 10, 25, 50, and 100 ng) for 2, 6, 24, and 48 h. As shown in Fig. 1, following 2 h of ST intoxication, cGMP could be identified in both T84 cell lysates and cell-free secretions. However, more cGMP could be found in the T84 cell lysates (Fig. 1A) than in secretions (Fig. 1B), especially at low ST intoxication doses. After 6 h of ST intoxication, there was still more cGMP in T84 lysates than in secretions, although it was beginning to become apparent that cGMP levels were decreasing in the lysates and increasing in the secretions. However, after 24 and 48 h of ST intoxication, approximately 10-fold more cGMP was found in the T84 secretions than lysates. These data suggest that intracellular accumulation of cGMP following ST intoxication occurs rapidly before cGMP is exported and reaches an equilibrium between 2 and 24 h after ST intoxication (Fig. 1B). Intracellular accumulation of cGMP is impaired by phosphodiesterase (PDE) activity, especially cGMP-specific PDE5 and PDE9A, which convert cGMP to GMP (18). Despite the addition of PDEis, intracellular cGMP levels decreased over time while secretory cGMP levels increased over time, suggesting that T84 intestinal epithelial cells may make a concerted effort to secrete cGMP upon ST intoxication (Fig. 1C). A summary of ST-induced cGMP production and export by T84 intestinal epithelial cells can be seen in Fig. 1D. This kinetic shift in cGMP localization could also help the development of ETEC-specific responses.

**FIG 2:**
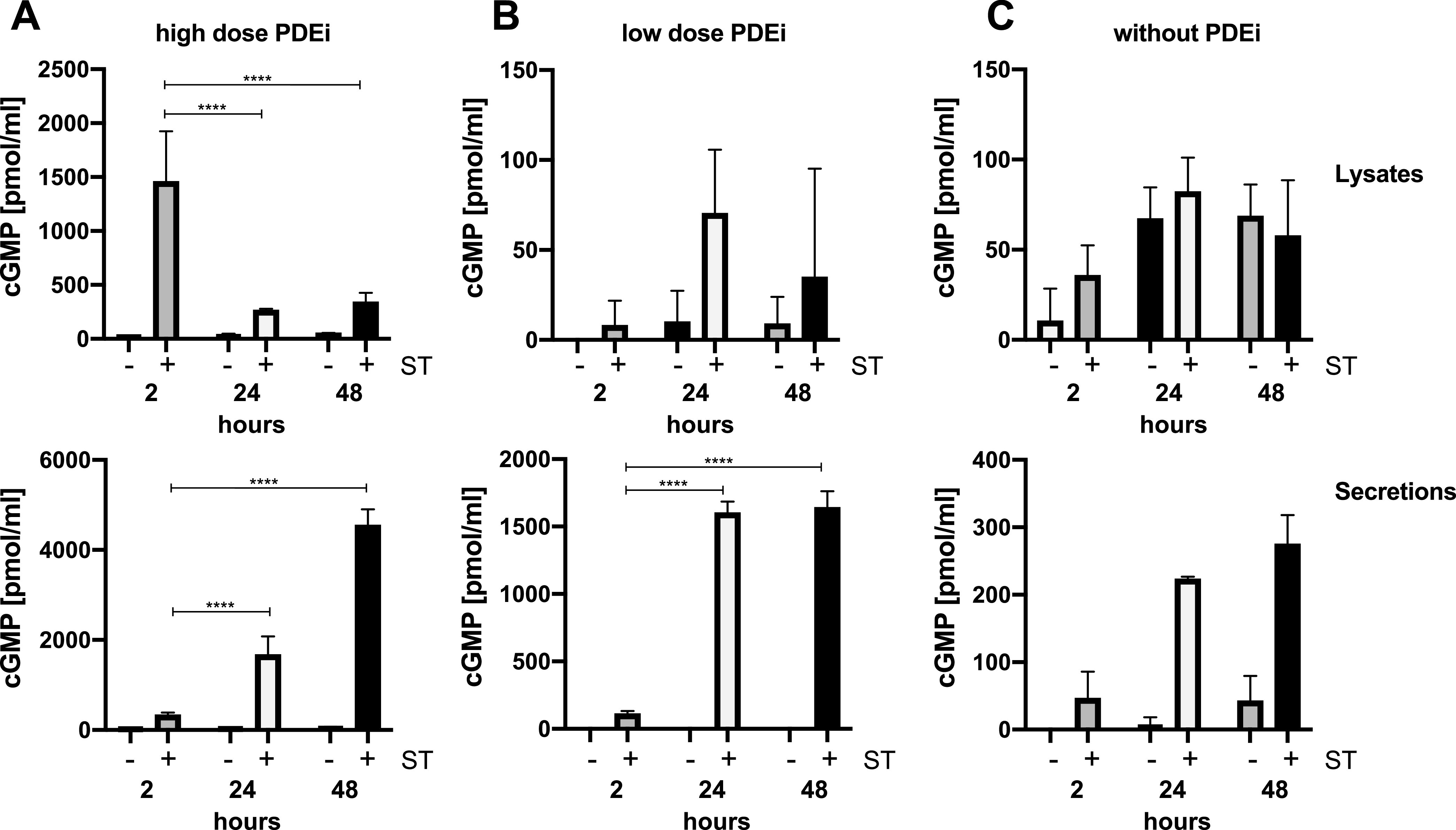
ST intoxication induces secreted cGMP in T84 epithelia in the absence of phosphodiesterase inhibitors. ST (50 ng) was applied to T84 cells for 2 h, 24 h, or 48 h with or without PDEis. Cells were pretreated for 1 h with full-dose zardaverine (20 μM) and vardenafil (30 μM) (A), 1/10-strength zardaverine (2.0 μM) and vardenafil (3.0 μM) (B), or no PDEis (C). As shown, inclusion of PDEis at full dose amplified the amount of cGMP that can be measured from T84 cell lysates after 2 h of ST intoxication. At 1/10-strength PDEis, the measurable cGMP from T84 lysates significantly plummeted; however, cGMP could still be measured in T84 secretions after extended intoxication. Moreover, ST-induced accumulation of cGMP could not be quantified in T84 lysates in the absence of PDEis, but cGMP could be measured in cell-free T84 supernatants. Data are averages from three independent experiments with three technical replicates per condition. Significance was determined using an ordinary one-way ANOVA. ****, *P* < 0.0001. Note that the *y* axis of each plot is scaled differently.

It is possible that induction of cGMP secretion following ST intoxication could be a function of inhibited cGMP turnover, since PDEis were also added to T84 cells. Prior studies have also used PDEis to describe the extracellular localization of cGMP as a function of ST treatment (11, 19). To assess the impact of exogenously added PDEis on induction of cGMP secretion in T84 cells following ST (50 ng) intoxication, we carried out experiments using three different PDEi concentrations: high-dose PDEis (20 μM zardaverine and 30 μM vardenafil), low-dose PDEis (2.0 μM zardaverine and 3.0 μM vardenafil), and no PDEis. When high-dose PDEis were used, 2 h ST intoxication stimulated high intracellular cGMP induction as measured in cell lysates, but low cGMP was measured in secretions (Fig. 2A). Following 24 or 48 h ST intoxication, cGMP was no longer measurable at high concentrations in lysates but was found in appreciable amounts in secretions. These data are comparable to the data shown in Fig. 1 despite different amplitudes of the cGMP signals, which is indicative of plate-to-plate variability. On the other hand, when low-dose PDEis were used, 50 ng ST failed to induce much measurable cGMP in lysates or secretions following 2 h intoxication (Fig. 2B). However, sustained 24 or 48 h ST intoxication allowed accumulation of secreted cGMP to levels that indicated that cGMP is being actively pumped out of T84 cells. (Fig. 2B). Finally, we showed that cGMP is secreted even in the absence of PDEis after 24 to 48 h ST intoxication (Fig. 2C). Note that the *y* axis of each plot (Fig. 2A to C) is scaled differently, likely due to the rapidity with which cGMP is broken down in the absence of PDEis. Our findings suggest that some ST-induced cGMP makes its way out of the cell into either the interstitial space or the intestinal lumen, where it cannot be accessed by intracellular PDEs for immediate isomerization. Confirmation of extracellular cGMP sets the stage for development of a more physiologically relevant understanding of ST pathogenesis and the role of secreted nucleotides as first messengers. We confirmed that polarized T84 monolayers secrete cGMP both apically and basolaterally following ST intoxication in the presence of PDEis but preferentially secrete cGMP basolaterally in the absence of PDEis (see Fig. S1 in the supplemental material). Together, these data suggest that that secreted cGMP could possibly modulate the epithelial-immune axis.

**FIG 3:**
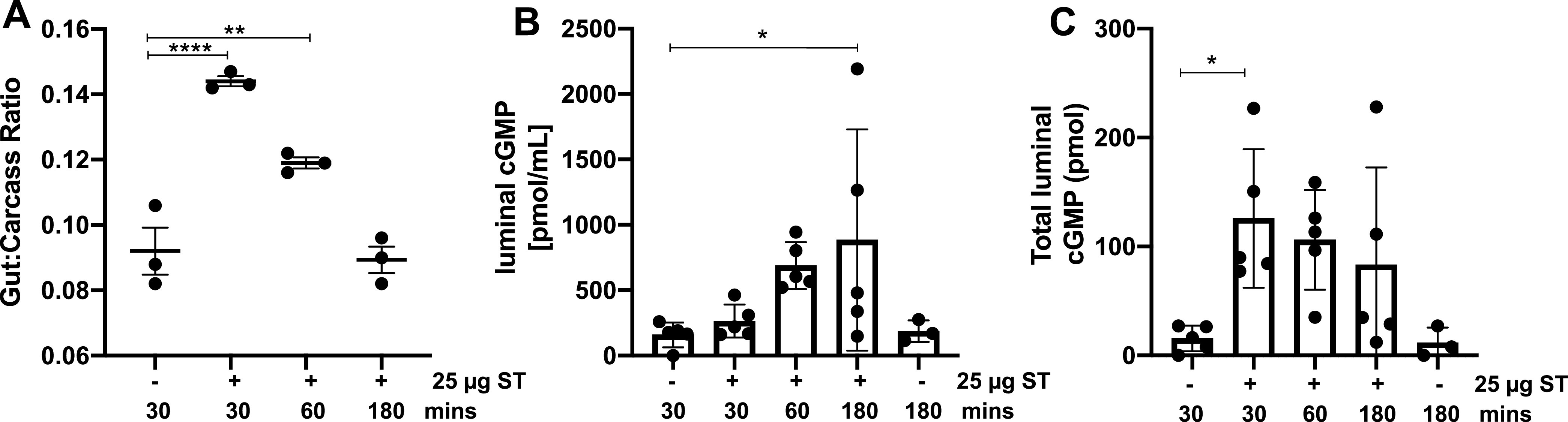
ST intoxication increases luminal cGMP *in vivo*. Six-week-old female BALB/c mice (*n* = 3 per group) fasted overnight and then were administered saline or ST (25 μg) by oral gavage. The animals were allowed to survive for 30 min, 1 h, or 3 h following exposure. ST intoxication induced a robust secretion after 30 min intoxication (A), as measured using the patent mouse assay. The secretory event induced by ST intoxication was also robustly resorbed, and no significant change to the animal’s gut-carcass ratio was seen after 3 h intoxication. ST exposure increases the luminal cGMP concentration as a function of time (B). Each animal’s luminal cGMP concentration was multiplied by the recovered luminal volume to reflect the total luminal cGMP changes as a function of ST intoxication (C). Data are from one experiment that was repeated three times. Significance was determined using an ordinary one-way ANOVA. *, *P* < 0.05; **, *P* < 0.01; ****, *P* < 0.0001.

Next, we turned our attention to the adult patent mouse assay (PMA) of ST enterotoxicity (3, 20) to determine if ST intoxication causes changes in luminal cGMP levels *in vivo*. We administered either saline or ST (25 μg) to 6-week-old BALB/c animals by oral gavage for 30, 60, or 180 min before euthanasia. As expected, ST intoxication induces rapid fluid accumulation, resulting in increases in the gut-carcass ratio compared to saline inoculation (3). Maximal ST-mediated secretion was observed after 30 min and waned as a function of time thereafter, returning to baseline at 180 min (Fig. 3A). Our results indicate that ST intoxication induces rapid fluid secretion that is quickly followed by rapid fluid reabsorption, since there were no differences in fecal output among the animal groups. We then assayed the small intestinal secretory fluid for cGMP and found that luminal cGMP concentrations increased as a function of ST intoxication time from 30 min to 180 min (Fig. 3B). Interestingly, the concentration of luminal cGMP was highest following 180 min of ST intoxication despite secretory fluid resorption (Fig. 3A). These data suggest that ST intoxication could potentially have longer-lasting effects via secreted cGMP than previously appreciated. Since the volume of secretory fluid changed as a function of ST intoxication time (i.e., the 30-min ST intoxication contained the most secretory fluid from luminal collections) (Fig. 3A), the concentration of luminal cGMP was multiplied by the volume of secretory fluid collected for each animal. We show that the total luminal cGMP for all ST treatment times was higher than the total luminal cGMP content for saline-inoculated controls (Fig. 3C). These data show that ST intoxication quickly induces the accumulation of luminal cGMP in animals, and our findings indicate that cGMP concentration changes rapidly in the ST-intoxicated gut, which may impact the longevity of ST ETEC pathogenesis or subsequent immune responses and cytokine signaling.

### ST intoxication alters inflammatory cytokine activity of intestinal epithelial cells.

**FIG 4:**
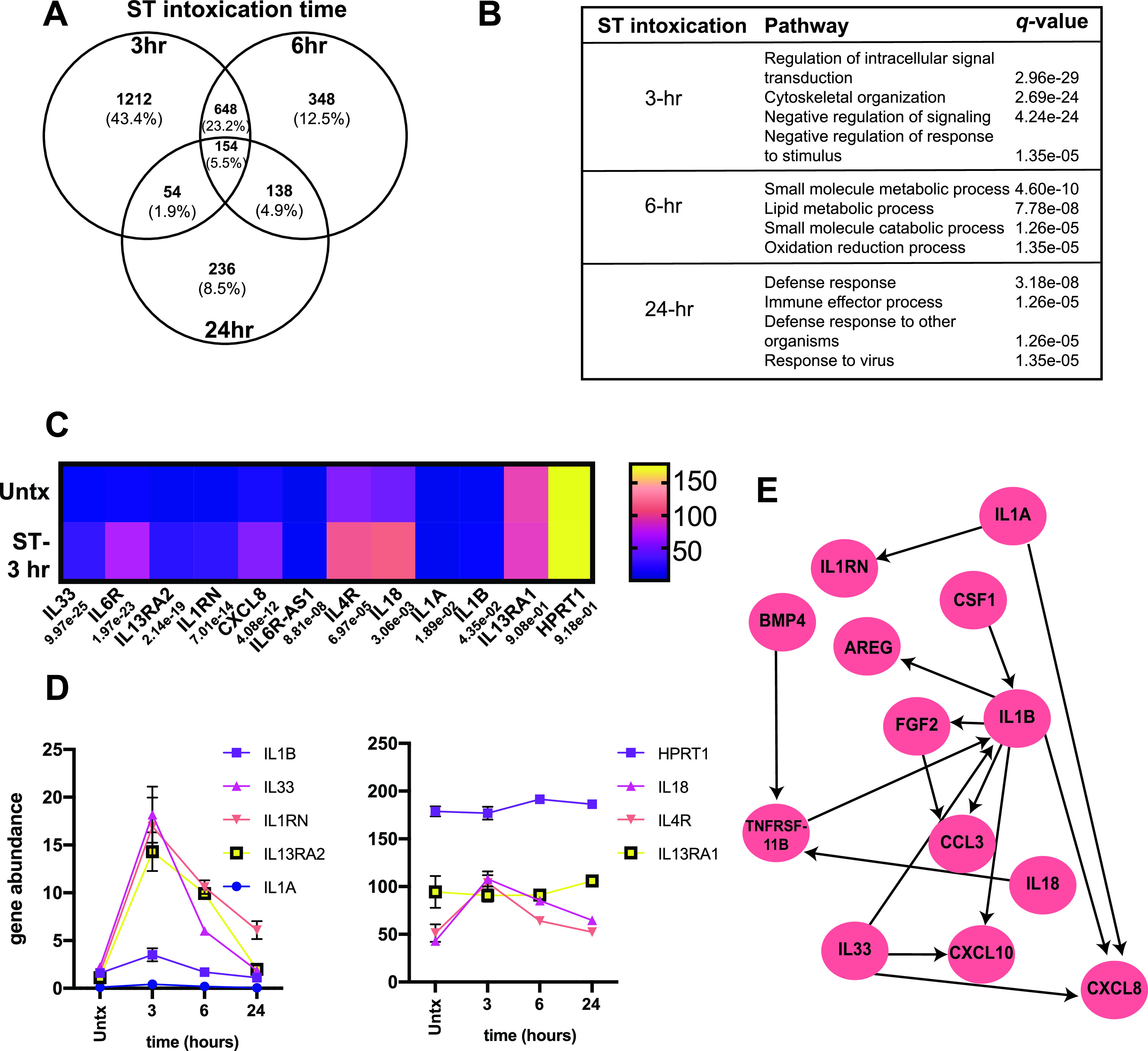
ST intoxication alters IL-1 family cytokine signaling in epithelial cells. ST (100 ng) was applied to T84 cells in the absence of any PDEi for 3, 6, and 24 h before RNA isolation for RNA-seq analysis. All genes that were differently expressed at *q* values of <0.5e−2 were included in this analysis. ST altered the expression of 2,068, 1,288, and 582 genes after 3, 6, and 24 h intoxication, respectively. (A) Venn diagram showing the number of genes that were differentially expressed at each time point. IL-1 family cytokine signaling pathways are modulated by 3 h ST intoxication. (B) Most significantly altered GO pathways in T84 cells as a function of 3, 6, and 24 h ST intoxication. (C) Heat map analysis displaying abundant IL-1 family and related inflammatory cytokines and genes in T84 cells as a function of 3 h ST intoxication. (D) Kinetics of the induction and return to baseline for IL-1 family and related inflammatory cytokines and genes in T84 cells as a function of ST intoxication. Coherent gene set analysis of the IL-1 superfamily shows the regulatory circuit following 3 h ST intoxication (D).

Previous studies have identified how ETEC infection alters the transcriptome of host genes using tissue culture cells and tissue explants (21). However, in an effort to better define the effect of ST intoxication and/or secreted cGMP on epithelial cells, we carried out a transcriptional study whereby RNA was isolated from T84 monolayers intoxicated with ST for 3, 6, and 24 h based on the results described above. ST intoxication significantly (*q *< 0.05) changed the expression of 2,068, 1,288, and 582 genes after 3, 6, and 24 h, respectively, out of a total of 18,996 genes confidently measured, compared to untreated controls (Fig. 4A; also, see Data Set S1). We found 648 genes in common following 3 h and 6 h ST intoxication, 138 genes in common following 6 h and 24 h ST intoxication, 54 genes in common following 3 h and 24 h ST intoxication, and 154 genes in common following 3 h, 6 h, and 24 h ST intoxication (Fig. 4A). Gene set enrichment analysis of GO pathways showed that the transcriptional response to ST changes over time. Interestingly, many members of the IL-1 family of cytokines were upregulated after 3 h ST intoxication, when the GO pathways for signal transduction and cytoskeletal organization are significantly changed. Prolonged (24 h) ST intoxication significantly altered the GO pathways of defense response and the immune effector process (Fig. 4B and C), suggesting that ST could alter the innate and adaptive immune response to ETEC that produces it.

The IL-1 family of cytokines plays a central role in inflammation and the response to infection. IL-1 signaling has been shown to be modulated by enteropathogens, including *Escherichia*, *Yersinia*, and *Shigella* (22). Prior studies have shown that members of the inflammatory IL-1 superfamily are induced in response to LT and ETEC (3, 23), so we sought to understand the impact of ST on the IL-1 family members. As shown by the heat map of transcriptional abundance (Fig. 4C), IL-33, IL-1β, IL-1ra, IL-1α, and IL-18 and other inflammatory targets and regulatory genes, including IL-13RA1, IL-13RA2, IL-6R, and IL-4R, were upregulated by 3 h ST intoxication. We focused on differentially regulated genes after 3 h ST intoxication, since ST induces rapid fluid secretion and luminal cGMP localization in animals, to understand if ST alters the epithelial transcriptome. Three-hour ST intoxication significantly upregulated the transcriptional abundance of genes encoding IL-33 (4.22-fold; *q *= 9.97e−25), IL-1β (1.65-fold; *q *= 4.35e−02), IL1RN (the gene encoding receptor decoy IL-1ra) (4.82-fold; *q *= 7.01e−14), IL-1α (2.30-fold; *q *= 1.9e−02), and IL-18 (1.85-fold; *q *= 3.06e−03) (Fig. 4D). Our data also show that ST treatment significantly upregulated IL-13RA2 (5.66-fold; *q *= 2.1e−19), IL-4R (1.62-fold; *q *= 7.0e−05), IL-6R (6.91-fold; *q *= 2.0e−23), and IL-6R-AS1 (6.5-fold; *q *= 8.81e−08) gene expression but not that of the gene encoding IL-13RA1 (1.0-fold; *q *= 9.1e−01). As shown in Fig. 4C, most IL-1 family members were significantly upregulated following 3 h ST intoxication, and their gene expression patterns returned to basal levels after 6 or 24 h ST intoxication. Only the IL1RN gene was significantly upregulated, compared to untreated cells, following 24 h ST intoxication. It is possible that acute inflammation could affect the development of long-term immunity. Coherent gene set analysis of the IL-1 superfamily showed the regulatory circuit following 3 h ST intoxication that matches with previous literature (Fig. 4E).

**FIG 5:**
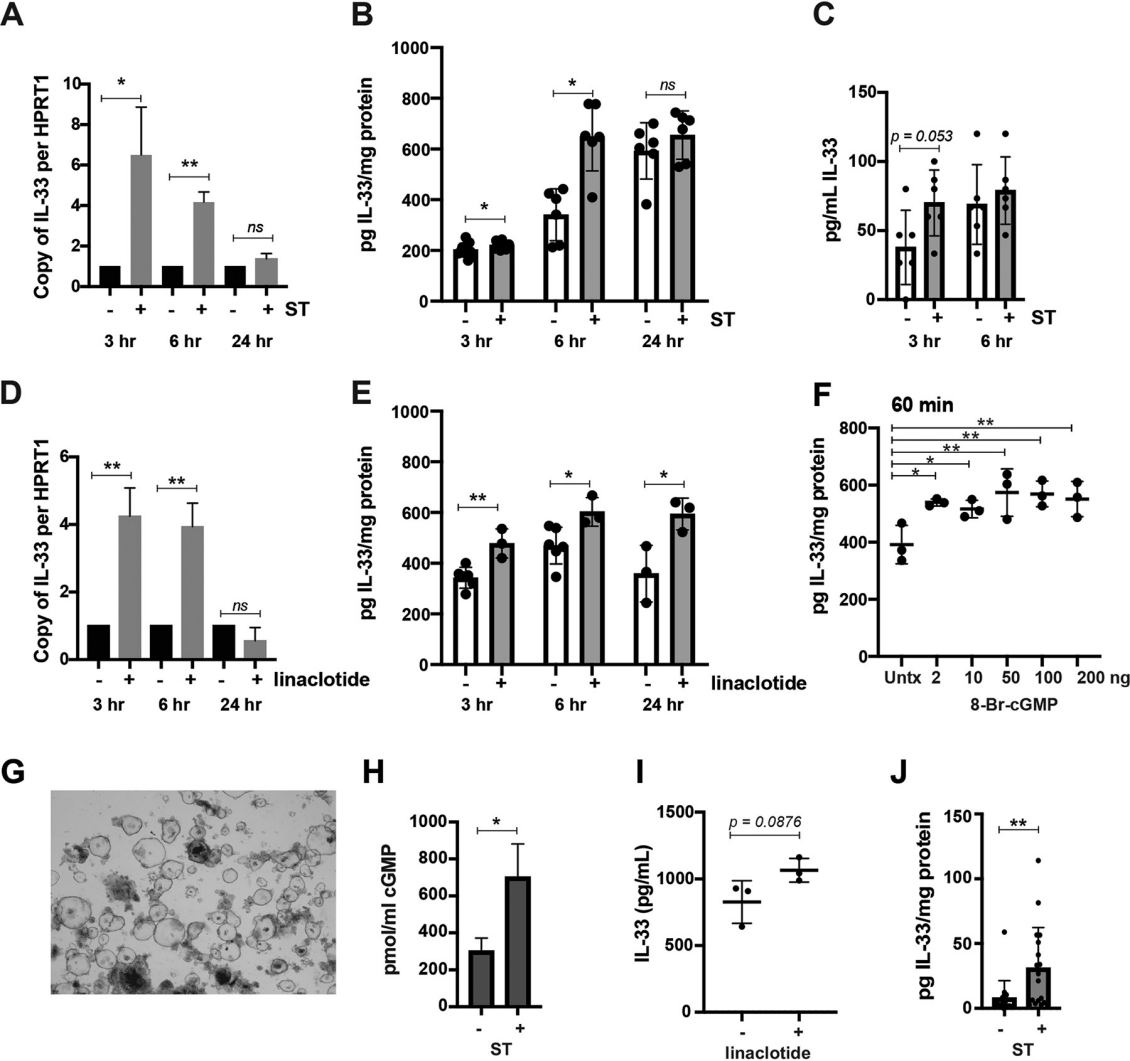
ST and linaclotide induce epithelial IL-33. (A) ST (100 ng) intoxication induced IL-33 transcripts as measured with qPCR. (B) ST (100 ng) induced IL-33 in T84 lysates after 3 and 6 h intoxication, compared to unstimulated cells. (C) ST (100 ng) induces IL-33 secretion from T84 cells after 3 h intoxication, compared to unstimulated cells. (D) Linaclotide (100 ng) induced IL-33 transcripts as measured with qPCR. (E) Linaclotide (100 ng) induced IL-33 in T84 lysates after 3, 6, and 24 h stimulation, compared to unstimulated cells. (F) Titration of 8-Br-cGMP on T84 cells for 1 h induced IL-33 from T84 lysates, compared to untreated cells. (G) Light micrograph (Nikon Eclipse TE300) of cultured human jejunal enteroids. (H) Human jejunal enteroids were stimulated with saline or ST for 3 h in the presence of PDEis. (I) IL-33 was measured in the human jejunal enteroid lysates after 3 h of saline or linaclotide stimulation. (J) IL-33 was measured in the mucosal scrapings of animals that were given saline or ST by gavage for 30 min. The data shown represent the aggregate of 3 independent experiments with at least 3 animals per group. qPCR data were normalized to copies of HPRT1 or ACTB. T84 experiments were carried out in the absence of PDEis. Data are from one experiment that was repeated three times. Significance for panels A to E and H to J was determined using a two-tailed *t* test comparing treated and untreated groups at each time point. Significance for panel F was determined using an ordinary one-way ANOVA. ns, not significant; *, *P* < 0.05; **, *P* < 0.01. Unless otherwise indicated, values are means and standard deviations.

IL-33 was the most significantly upregulated IL-1 family cytokine after 3 h ST intoxication based on its Wald test *q* value following transcriptome analysis via RNA sequencing (RNA-seq). IL-33 has been shown to promote T_H_2 responses and intestinal repair (24, 25) following acute infection. Moreover, IL-33 binds to CD4^+^ ST2^+^ IL-17A^+^ T_H_17 cells and suppresses IL-17A production, essentially converting them to transient regulatory T cells (T_reg_s) (26). Since our ST came from highly purified bacterial supernatants, we first set out to verify that ST and not low levels of endotoxin were responsible for transcriptional upregulation of IL-33. The first indication that endotoxin was not the cause of ST-mediated induction of IL-33 and other IL-1 family cytokines was found in the RNA-seq data sets, which showed that ST intoxication does not induce tumor necrosis factor alpha (TNF-α) in T84 cells despite the abundance of TLR4 (see the supplemental material). We detoxified ST using standard Detoxi-Gel columns and also sought to confirm the induction of IL-33 with the FDA-approved ST analogue linaclotide. Linaclotide is an ST mimic commonly prescribed for patients with irritable bowel syndrome with constipation (IBS-C) and is devoid of endotoxin, since it is manufactured via solid-phase synthesis. We treated T84 cells side by side with ST and linaclotide for 3, 6, and 24 h and analyzed both the changes in IL-33 gene expression via quantitative PCR (qPCR) using the gene for hypoxanthine-guanine phosphoribosyltransferase (HPRT1) as a housekeeping gene (27) and IL-33 protein expression via ELISA. Previously, it was shown that cAMP-activating agents synergize with LPS to induce IL-33 via CREB in murine macrophages that could be suppressed with using a protein kinase A inhibitor, H-89 (28). As shown for ST in Fig. 5A and for linaclotide in Fig. 5D, both treatments significantly upregulated IL-33 gene expression following 3 and 6 h intoxication. Neither ST nor linaclotide induced IL-33 gene expression after 24 h intoxication. Moreover, as shown for ST in Fig. 5B and for linaclotide in Fig. 5E, IL-33 protein levels were induced as a function of ST intoxication for 3 and 6 h and linaclotide intoxication for 3, 6, and 24 h. Importantly, we also show that there was a tendency for IL-33 to increase in T84 cell culture supernatants following ST intoxication (Fig. 5C), though to our knowledge IL-33 was not released by apoptosis, as lactate dehydrogenase (LDH) levels in the supernatants were not detected (Fig. S2).

We then sought to determine if IL-33 could be induced by cGMP via application of the cell-permeative cGMP analog 8-Br-cGMP to T84 cells. Lysates of T84 cells treated with 8-Br-cGMP (100 ng) contained significantly more IL-33 after 60 min (Fig. 5F) of treatment. To confirm the physiological relevance of our findings, we also showed that ST applied to the apical surfaces of polarized human jejunal monolayers induced cGMP and that the IL-33 recovered in human jejunal monolayer lysates tends to increase upon linaclotide treatment (Fig. 5G to I); it should be noted that ST intoxication did adversely affect cell viability (Fig. S2). Linaclotide is approximately 4-fold less toxic than native ST on a per-mass basis, and it is possible that extended linaclotide prolongs GC-C stimulation and IL-33 expression.

We next looked for ST-induced IL-33 production *in vivo*. As shown in Fig. 5J, small intestinal lysate samples collected from 6-week-old BALB/c females treated with ST for 30 min showed significantly increased IL-33 production relative to saline-treated animals. Lastly, intestinal tissue from the ileum and colon of ST-treated animals appeared elongated and swollen upon hematoxylin-and-eosin (H&E) staining and had less prevalent phalloidin staining from immunofluorescent micrographs, compared to unintoxicated tissue, supporting our RNA-seq data (Fig. S3) and previous observations that ST intoxication alters cytoskeletal pathways (29). Unlike ST-mediated cGMP induction of IL-33, we showed that ST treatment but not 8-bromo-cGMP treatment induced IL-8 in T84 cells. This finding shows that ST-mediated IL-8 production in T84 intestinal epithelial cells requires more complex signaling events than the increase in cyclic nucleotide concentrations (Fig. S4).

### ST modulates mucosal immune responses following parenteral and oral immunization.

**FIG 6:**
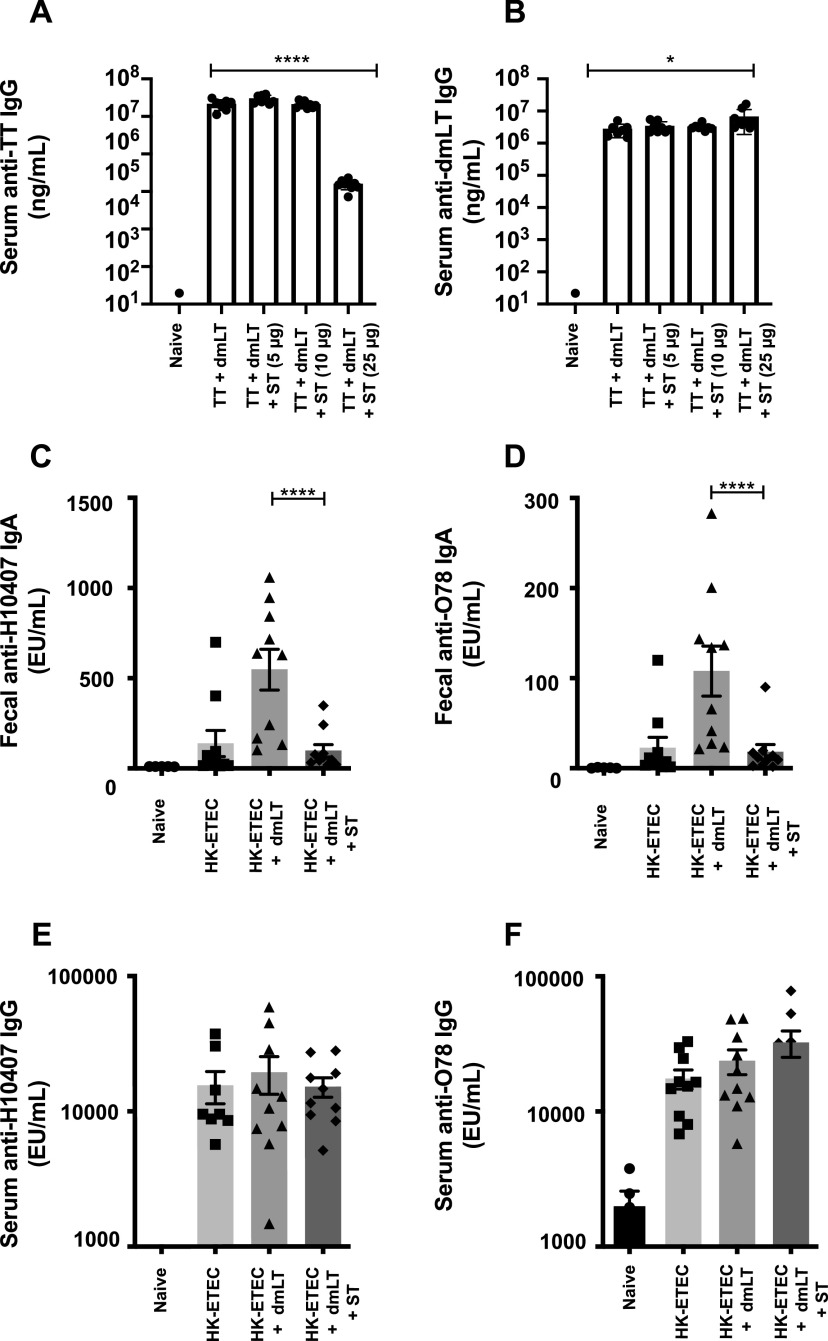
ST alters antibody development following parenteral and mucosal immunizations. ST was titrated (0, 5, 10, and 25 μg) into intradermal immunization formulations containing tetanus toxoid (TT) (10 μg) and dmLT (1.0 μg). The immunizations were carried out twice, 14 days apart, and the sera and feces were analyzed for antigen-specific IgG1 and IgA, respectively. ST (25 μg) suppressed the induction of serum anti-TT IgG (A) without affecting the induction of serum anti-dmLT IgG (B). ST (25 μg) was added to oral immunization formulations containing heat-killed ETEC H10407 (0.5e10 CFU/dose) and dmLT (25 μg). Naïve animals received an oral gavage consisting of saline. The immunizations were carried out twice, 21 days apart, and the sera and feces were analyzed for antigen-specific IgG1 and IgA, respectively. ST (25 μg) suppressed the induction of fecal anti-H10407 IgA (C) and fecal anti-O78 LPS IgA (D) without affecting the induction of serum anti-H10407 IgG (E) or serum anti-O78 IgG (F). The fecal IgA data were calibrated per unit of fecal mass. Values are means and standard errors of the means. The data are from one experiment that was repeated three times. Significance was determined using a one-way ANOVA with the Bonferroni posttest for all groups compared to the naive group. *, *P* < 0.05; ****, *P* < 0.0001.

LT and LT-based adjuvants, including dmLT, have been used to induce stronger humoral immune responses (5, 6). Thus, we wanted to see if ST has a direct effect on adaptive immunity by examining the immune response to orally administered LT-based antigens and the heterologous antigen tetanus toxoid (TT). In lieu of LT, we chose to use the LT-based detoxified adjuvant dmLT to avoid the complication of intestinal secretion. We immunized animals intradermally on days 0 and 14 with either saline, TT (10 μg) plus dmLT (1.0 μg), or TT plus dmLT in the presence of increasing quantities of ST (5, 10, or 25 μg). We chose the maximal dose of ST (25 μg) based on our experience using the patent mouse model to induce fluid secretion into the lumen of a 6-week-old animal (Fig. 3A). Formulations containing TT and dmLT with ST (5 and 10 μg) induced similar serum anti-TT IgG1 (Fig. 6A) and serum anti-dmLT IgG1 levels (Fig. 6B). However, inclusion of 25 μg ST in formulations containing TT and dmLT suppressed the serum anti-TT IgG1 production (Fig. 6A) without affecting serum anti-dmLT IgG1 response (Fig. 6B) compared to animals immunized with TT and dmLT only.

Finally, we wanted to determine the impact of ST on immune responses following oral exposure to ETEC antigens. For these studies, we immunized BALB/c mice orally with heat-killed ETEC H10407 at 0.5e10 CFU/dose in the presence or absence of dmLT or ST. The enterotoxin profile of H10407 includes LT, STp, STh, and the colonization factor antigen is CFA/I. ETEC H10407 was heat killed at 60°C for 1 h prior to inoculation using an oral immunization strategy containing two oral exposures on day 0 and day 21. Inoculum contained heat-killed ETEC strain H10407 (0.5e10 CFU/dose) with or without dmLT (25 μg) and ST (25 μg) added exogenously. Again, dmLT was used for these studies to overcome the cofounding variable of fluid secretion induced by native LT. Our data show that ST suppressed the induction of secretory IgA to both whole-cell ETEC H10407 (Fig. 6C) and O-78 lipopolysaccharide (LPS) (Fig. 6D) antigens following oral immunization, but there was no impact on the development of circulating anti-ETEC (Fig. 6E) or anti O78 (Fig. 6F) IgGs. Moreover, when LT holotoxin was used, ST also suppressed anti-H10407 and anti-O78 antibody production (Fig. S5). Inclusion of ST did not suppress the development of anti-dmLT IgA or the development of serum anti-dmLT IgG antibodies (data not shown). Moreover, ST-mediated suppression of antigen-specific fecal IgA appears to be dependent on ST activity, since ST toxoid A14T failed to decrease production of antibody to ETEC H10407 and O78 LPS antigens, compared to both STh and STp (Fig. S6).

## DISCUSSION

ST-producing ETEC is among the top four pathogens in children aged 0 to 60 months with moderate to severe diarrhea (30, 31). Approximately 73% of wild ETEC strains encode and produce both LT and ST (2). One gap in the knowledge regarding ETEC pathogenesis concerns the evolutionary advantage for ETEC to maintain two enterotoxins—LT and ST—that, at surface level, perform the same function of causing secretory diarrhea. Along the same vein, we asked ourselves if maintenance of two enterotoxins is related to the well-documented phenomenon of children under the age of 5 years being unable to develop long-term protection against ETEC infection. Understanding these two problems and how they work in conjunction has the potential to influence ETEC vaccine development.

In our studies, we utilized a combination of *in vitro* tissue and enteroid culture and *in vivo* PMAs to support the notion of an alternative function of ST beyond enterotoxicity. A dual role of a bacterial toxin is not a new phenomenon. For example, Vibrio cholerae has been shown to upregulate genes related to iron acquisition only when cholera toxin is produced, thereby acting as a diarrheagenic agent as well as an important factor for nutrient uptake (32). Additionally, recent data from our lab showed that ST is an iron- and zinc-binding peptide that is detoxified by mucosal metallothionein (20), potentially opening a new line of inquiry for diarrheagenic toxins, luminal transition metal homeostasis, and redox balance. Moreover, LT has been shown to inhibit intestinal antioxidant ascorbic acid uptake in Caco-2 cells (33), potentiating cells to be less efficient at modulating oxidative stress. The study we present here suggests that ST, via epithelium-derived IL-33, could polarize the immediate mucosal immune response and partially explains why children do not develop robust, long-lasting immunity to ETEC antigens when infected with ST^+^ ETEC.

Previously, it was shown that oral gavage with 8-Br-cGMP evoked secretion in the infant mouse model (34), alluding to the existence of functionally active cGMP in the intestinal lumen. Here, we show that that ST-induced cGMP is secreted by both T84 intestinal epithelial cells *in vitro* and murine epithelial cells *in vivo*. cGMP is traditionally described as a second messenger that modulates cKGII serine/threonine kinase (35) and PKA (35) signaling cascades. However, our data suggest that cGMP could be a host paracrine mediator in response to infection. Indeed, apical application of the purinergic agonist UTP triggers calcium-dependent chloride secretion from bronchial epithelia (36). Furthermore, recent studies of HDM-induced inflammation showed that intranasal administration of HDM and cyclic GMP-AMP (cGAMP) promoted IL-13 and IL-5 production from lymph node cells dependent on IL-33 expression in lung fibroblasts and eosinophil infiltration into bronchioalveolar lavage fluid (12), suggesting that cyclic dinucleotides have the ability to activate innate lymphoid cells 2 (ILC2s) and promote T_H_2 responses.

Our RNA-seq data show that ST significantly alters the transcriptome of T84 epithelial cells, and ST intoxicates cells by altering signaling pathways GC-C activity to induce cGMP. Recent reports suggested that PDE5 restricts accumulation of intracellular cGMP during ST-ETEC infection (11), but our RNA-seq data show that cGMP-specific PDE9 is upregulated (1.66-fold; *q *= 1.38e−05), while PDE5 is downregulated (2.1-fold; *q *= 3.24e−02) in T84 cells following 3 h ST intoxication (see the supplemental material), suggesting that the response to acute changes in cGMP concentrations is more complex than previously described. Of note, ST modulates host genes involved in cytoskeletal remodeling and cellular locomotion, implying that secreted ST modifies the epithelial actin cytoskeleton, in ways that are similar to those employed by enteropathogenic Escherichia coli (EPEC) and enterohemorrhagic Escherichia coli (EHEC) (15). Of particular interest to us was that ST intoxication enhanced production of the IL-1 family member IL-33, an alarmin released upon epithelial cell damage (25) and implicated in T_H_2-mediated inflammation, including antihelminth immunity (37), epithelial repair (38), allergic asthma (39), goblet cell hyperplasia (40), and inflammatory bowel disease (41). IL-33 may be a key cytokine involved in innate and adaptive immune responses following exposure to ETEC. In fact, the adherent and invasive E. coli isolate LF82 promotes intestinal fibrosis via IL-33 and ST2 signaling in a *Salmonella* infection model (42).

IL-33 binds to the heterodimeric receptor of ST2 and IL-1RAcP and on many different cell types (40). IL-33 treatment of lamina propria lymphocytes induces the transcription of IL-1rl1, Gata3, IL-13, IL-4, IL-31, and Retnlb genes, effectively polarizing to a T_H_2 response (43). IL-33, in the presence of IL-25, potentiates ILC2 populations to produce IL-13 to promote intestinal remodeling to facilitate infection resistance with helminths and parasites (44). Importantly, IL-33 suppresses intestinal T_H_17 responses and promotes immunosuppressive T_reg_ phenotypes (26, 45).

ST-mediated induction of IL-33 was found not only in T84 epithelial cells but also in small intestinal enteroid cultures and in animals. We have not determined how IL-33 is released or even if it is released in animals. However, we show that IL-33 is induced in lysed mucosal scrapings shortly after ST intoxication. IL-8 was secreted from T84 cells at high levels following ST exposure, but as previously shown, addition of ST does alter LT-induced IL-8 levels (3), suggesting that IL-8 induction occurs independent of cyclic nucleotides but possibly via inducible nitric oxide synthase (20), which is further supported by our data in this study.

Moreover, we show that ST intoxication induced expression of the IL-1 decoy receptor IL-1ra in T84 epithelial cells, and functional adaptation of T_reg_s has been shown to be dependent on IL-1 family receptors (45). Future studies are needed to define the role of increased epithelial IL-1ra production on the development of mucosal immune responses. Our data also show that ST intoxication of T84 epithelial cells induces expression of the IL-13RA2 gene (Fig. 4C and D), which encodes the decoy receptor for IL-13, and IL-4R (Fig. 4C and D), which can bind both IL-13 and IL-4. It is not yet known if IL-13RA2 and IL-4R expression is induced by ST, ST-triggered cGMP, or ST-triggered IL-33. Based on our data, however, it is possible that ST induction of IL-33 could induce IL-13 via ILC2s and compete with IL-4 for IL-4R, thereby affecting B cell antibody responses and contributing to the decreased anti-ETEC response we see in animals immunized with ST. Experiments are under way to determine the effects of ST and/or the effects of the ST-inflamed epithelium with enteric lamina propria lymphocytes, including B cells.

IL-33 has been shown to promote intestinal IgA responses in the colon (46), which may appear contrary to the results presented here. It should be mentioned that our results show that ST induces IL-33 in small intestinal tissue, not in colonic tissue. Moreover, LT has been shown to induce T_H_17 responses (6), and IL-33 controls the T_H_17-to-T_reg_ balance in the small intestine (26, 45), suggesting that there is differential control over immune responses in different tissue types. We found that the addition of ST to immunization formulations blunted antibody development in models using both heterologous and ETEC-based antigens. Based on our findings, we hypothesize that ST, in addition to acting as a fast-acting enterotoxin, quickly reprograms small intestinal epithelial cells to produce and secrete IL-33, which short circuits host immune response development and potentiates subsequent enteric reinfection. Perhaps this phenomenon illustrates an evolutionary advantage the ST^+^ LT^+^ ETEC strains have over LT^+^ or ST^+^ ETEC strains, in that they can subvert host immune responses. This is perhaps a reflection of the fact that ST and LT are frequently produced together during active ETEC infection. Interestingly, the inclusion of ST (5 or 10 μg) did not alter the magnitude of serum anti-TT IgG1, suggesting that ST threshold during infection could be a contributing factor that alters anti-ETEC immune responses. Since no one truly understands the concentration of ST in the intestinal lumen or how it is delivered to epithelial cells, such thresholds may exist. These findings may help explain why children do not develop natural immunity to ETEC after infection and reinfection until after age 5. Moreover, IL-33 and ILC2 levels are higher in the lungs of neonatal animals (47). Based on our data identifying IL-33 as an immunosuppressant, this phenomenon is worth investigating in the gut; IL-33 neutralization may be important for inducing broad anti-ETEC immunity. It is worth noting that the development of immune responses to ETEC varies depending on dose and isolate (48); however, existing data suggest that antibody production against LT^+^ ETEC strains does not necessarily protect against infection from a heterologous LT^+^ ETEC strain (14).

One important point to address is that our PMA and subsequent readouts are based only on the use of purified ST and therefore may not place ETEC infection into the proper context with the bacterium and other virulence factors, including LT. Infection of animals with ETEC (ST^+^ ETEC, LT^+^ ETEC, and ST^+^ LT^+^ ETEC) itself may provide more nuance with regard to cGMP secretion and cytokine production. Moreover, the amount of ST produced and secreted during an active ETEC infection is unknown, and the juxtaposition of ETEC cells on intestinal epithelium may further modulate immune responses.

In short, there is still much to be learned about ST and its effects on the host epithelium and immune system. We have described a system in which ST modulates the host epithelial transcriptome in a way that diverts the immune response away from productive antibody production. Our belief is that this diversion of the immune response is in part responsible for recurrent ETEC infections in children younger than 5 years. Continuing this line of investigation will lead to further insight about ST pathogenesis to inform rational vaccine design.

## MATERIALS AND METHODS

### Animals.

Female BALB/c mice (6–8) weeks were purchased from Charles River Laboratories (Wilmington, MA). All of the procedures were approved by the Institutional Care and Use Committee at Tulane University School of Medicine (protocol 768).

### Human subjects.

Human intestinal biopsy specimens were collected postmortem, in collaboration with surgical residents associated with the Louisiana Organ Procurement Agency (LOPA). Intestinal biopsy specimens were collected only after informed consent had been obtained from the next of kin regarding the use of tissue for research applications. Title 32 of the Code of Federal Regulations (219.102 f) defines human subject research as coming from a living subject, so our use of intestinal organoids is not considered human research. All of the procedures were approved by the Institutional Review Board at Tulane University School of Medicine (protocol 2019-047).

### Enterotoxins and cell lines.

ST was purified from supernatants of recombinant strain 9115 as described previously (20). ST purified using this method has been deposited in BEI Resources under the catalogue numbers NR-50760, NR-50761, NR-50762, NR-50763, and NR-50764, and NR-50765. ST mutant A14T was purified using similar procedures. Linaclotide was dissolved in phosphate-buffered saline (PBS), and peptide concentration was determined and adjusted to a 1.0-mg/ml stock. 8-Br-cGMP was from Cayman Chemicals (no. 15992). T84 cells (CCL-248) were purchased from ATCC and were maintained and cultured on 24-well tissue culture-treated plates or on Transwell inserts as previously described (20). Tetanus toxoid was purchased from Statens Serum Institut (Copenhagen, Denmark). LTh and dmLT were purified as previously described (5).

Human intestinal enteroids were derived from intestinal crypts isolated from the jejunum of postmortem donors based on previously published methods (49). To make polarized monolayers of human intestinal epithelial cells, 3D spheroids were recovered from Matrigel (Corning) and dissociated with 10 mM EDTA in Hanks balanced salt solution (HBSS). The spheroids were then further dissociated by pipetting. The cells were resuspended in human IntestiCult organoid growth medium (Stemcell no. 06010) containing 10 μM Y-27632 (Tocris no. 1254) and 10 μM CHIR99021 (Tocris no. 4423) and plated on collagen-coated (Sigma no. C5533-5MG) Transwell inserts (Corning no. 3470). The following day, medium was replaced with human IntestiCult organoid growth medium without Y-27632 and CHIR99021 until confluence was reached via monitoring of transepithelial resistance (TER) (3).

### cGMP assay.

T84 monolayers in 24-well, flat-bottom cell culture plates were grown to 80% confluence. Polarized T84 monolayers were grown on collagen-coated (Sigma no. C5533-5MG) Transwell inserts (Corning no. 3470), and TER was measured daily (3). Wells were used in experiments only after the TER measured >1,000 Ω · cm^2^. One-hour prior to ST intoxication, cells were incubated with or without the PDEis zardaverine and vardenafil for 2, 6, or 24 h. Cell lysates and cell-free supernatants were diluted 1:20 and assayed for intracellular and secreted cGMP, respectively, using a cGMP Parameter assay kit (R&D Systems no. SKGE003) according to the manufacturer’s instructions, as previously described (3, 20). Luminal secretions from PMA experiments were also subjected to cGMP determination.

### Patent mouse model.

Adult patent mouse assays (PMAs) were conducted on 6-week-old BALB/c mice from Charles River Laboratories. Female mice were used in PMA experiments, as they were used in previous studies to establish this model (3). The mice were given saline or toxin (either LT or ST) via gastric lavage using a bent 20-gauge feeding needle. Following toxin administration, adult mice were incubated for 30 min, 1 h, or 3 h at room temperature. The mice were sacrificed by CO_2_ inhalation and cervical dislocation. The entire intestine from the duodenum to the rectum was removed, the gut-to-carcass ratio determined, and the luminal secretions collected. The metric of the PMA is the ratio of the animal’s gut weight to the animal’s carcass weight, with increased fluid measured as weight in the gut lumen compared to the remaining carcass weight. The mice were maintained on a standard laboratory diet, but food was denied for 18 h prior to enterotoxin experiments, while water was allowed *ad libitum*.

### Cytokine/chemokine measurements.

T84 cell lysates or secretions were collected following saline, ST, or linaclotide administration. Samples were clarified, and protein content was determined using the bicinchoninic acid (BCA) method (Thermo Fisher no. 23225). Samples were applied to IL-33 DuoSet ELISAs (R&D Systems) according to the manufacturer’s instructions. Experiments were repeated at least three times. Cytokine quantities are plotted per unit mass for cell lysates and total picomoles per milliliter for secretions.

### LDH assay.

Supernatants from treated cells were collected and assessed for the presence of LDH using the Pierce LDH cytotoxicity assay kit (Thermo Scientific no. 88953) in accordance with the manufacturer’s protocols. Data are reported as percent lysis by taking the ratio of the absorbance (490 nm) of cell culture supernatants to either the absorbance (490 nm) of lysed T84 cells or the absorbance (490 nm) of the positive control provided in the kit.

### RNA isolation and qPCR.

T84 cells were grown to 80% confluence on 24-well tissue culture-treated plates. T84 cells were intoxicated with ST or linaclotide for 3, 6, and 24 h, followed by RNA extraction with a Qiagen RNeasy kit. RNA isolated from untreated T84 monolayers served as the control. RNA was quantified using a NanoDrop C instrument, and 1.0 μg RNA was reverse transcribed with and without reverse transcriptase (Bio-Rad Inc.). qPCR was carried out using PrimeTime qPCR probes from IDT, including the following: for IL-33, Hs.PT.58.21416460; for HPRT1, Hs.PT.58v.45621572; and for ACTB, Hs.PT.39a.22214847. Reactions were carried out with PrimeTime gene expression master mix (IDT) on a CFX Connect system (Bio-Rad). We did not see genomic DNA contamination of our RNA samples, as tested by performing qPCRs on RNA samples without reverse transcriptase. Transcript levels were calibrated to the housekeeping gene for HPRT1 or ACTB. Changes in mRNA expression were determined using the comparative cycle threshold (*C_T_*) method.

### RNA-seq.

Total RNA from T84 cells (1 to 4 μg) was used as starting material for deep sequencing using the Illumina TruSeq RNA sample preparation guide, v2. Briefly, mRNA was purified with oligo(dT) beads, fragmented with magnesium and heat-catalyzed hydrolysis, and used as a template for first- and second-strand cDNA synthesis with random primers. The cDNA 3′ ends were adenylated, followed by adaptor ligation and a 15-cycle PCR to enrich DNA fragments. Quantification of cDNA libraries was performed by using a Kapa Biosystems primer premix kit with Illumina-compatible DNA primers. The cDNA libraries were pooled at a final concentration 1.8 pM. Single-read sequencing was performed on an Illumina Genome Analyzer IIx and NextSeq 500.

### Intradermal immunizations.

Intradermal immunizations were carried out in 6- to 8-week-old female BALB/c animals with 5 animals per immunization group. The animals were grouped as follows: group 1, naive; group 2, TT (10 μg) with dmLT (1.0 μg); group 3, TT (10 μg) with dmLT (1.0 μg) and ST (5 μg); group 4, TT (10 μg) with dmLT (1.0 μg) and ST (10 μg); group 5, TT (10 μg) with dmLT (1.0 μg) with ST (25 μg). The animals were intradermally immunized with formulations of no more than 50 μl twice, 14 days apart. The animals were humanely euthanized by CO_2_ inhalation followed by cervical dislocation. Serum was collected via cardiac puncture, and fecal pellets were collected from colonic contents. Antigen-specific fecal IgA levels were calibrated to total fecal mass. All procedures were approved by the Tulane University School of Medicine Institutional Animal Care and Use Committee (IACUC).

### Heat-killed-ETEC immunizations.

ETEC H10407 was heat killed by incubation at 60°C for 1 h. Aliquots were plated on LB agar to ensure 100% killing. Oral immunizations were carried out in 6- to 8-week-old female BALB/c animals with 8 to 10 animals per group. The animals were orally immunized with a 20-gauge feeding tube two times 21 days apart with saline (naive), heat-killed H10407, heat-killed H10407 with dmLT (25 μg), or heat-killed H10407 with dmLT (25 μg) and ST (25 μg). Heat-killed ETEC was delivered at 0.5e10 CFU per dose. In some experiments, dmLT was replaced with LT holotoxin and ST was replaced with the ST mutant toxoid ST-A14T. The animals were humanely euthanized by CO_2_ inhalation followed by cervical dislocation. Serum was collected via cardiac puncture, and fecal pellets were collected from colonic contents and weighed. All procedures were approved by the Tulane University School of Medicine IACUC.

### ELISAs.

Flat-bottom ELISA plates were coated with IgG1 or IgA standard and capture antigen (0.1 μg/well) overnight at 4°C. Plates were washed three times using 0.5% PBS-Tween (PBS-T) and blocked with 2% milk in PBS-T for 1 h at room temperature. Plates were washed three times using PBS-T before addition of fecal or serum samples. On the day of sample collection, fecal pellets were homogenized in fecal buffer (PBS, Tween 20, EDTA, and a protease inhibitor cocktail [Roche no. 11836153001]). Fecal homogenates were centrifuged at 3,000 rpm, and supernatants were collected and stored at 4°C until use. Fecal samples were used neat or diluted 1:2 in 0.2% milk in PBS-T and diluted 1:2 down each plate. Serum samples were diluted 1:50 in 0.2% milk in PBS-T and diluted 1:4 down each plate. Plates were incubated overnight at 4°C. Plates were washed three times with PBS-T. Secondary antibody was diluted in 0.2% milk in PBS-T: anti-mouse IgG-alkaline phosphatase (AKP; Sigma no. A1902) was diluted 1:1,000, and horseradish peroxidase (HRP)-conjugated anti-mouse IgA (Southern Biotech no. 1040-05) was diluted 1:2,500. Plates were incubated in secondary antibody for 1 h at room temperature. Plates were washed three times with PBS-T. Fecal ELISAs were developed using two-part peroxidase substrate (Seracare no. 5120-0047). Serum ELISAs were developed using diethanolamine and 4-nitrophenyl phosphate disodium salt hexahydrate tablets (Sigma no. N2765). Readouts were achieved using an Epoch2 microplate reader at 405 nm for serum ELISAs and 650 nm for fecal ELISAs. Antigen-specific fecal IgA levels were calibrated to total fecal mass.

### Immunofluorescence.

Tissue sections were flushed with PBS and flash frozen in OCT medium using dry ice and ethanol. Tissue was cut into 4-μm sections using a Leica CM1860 cryostat. Tissue sections were used for in-house immunofluorescence (IF) staining or sent to the Tulane histology core for H&E staining. For IF staining, each section was fixed in 4% phosphate-buffered formaldehyde and incubated at room temperature for 10 min. Slides were washed three times in cold PBS and then permeabilized for 10 min at room temperature using 0.1% Triton X-100 solution. Slides were washed three times with PBS. Wells were blocked for 1 h at room temperature using blocking solution (2% normal goat serum, 1% bovine serum albumin [BSA], and 0.05% NaN_3_ in PBS). Conjugated primary antibodies (phalloidin-conjugated A488 [Thermo Fisher no. A12379] and wheat germ agglutinin-conjugated A594 [Thermo Fisher no. W11262]) were diluted 1:80 and 1:2,000, respectively, in blocking solution and incubated with each slide for 1 h at room temperature. Slides were washed three times with PBS, protected from light, and mounted using MOWIOL 4-88 with glycerol and 2.5% Dabco mountant with 300 nM DAPI (4′,6-diamidino-2-phenylindole; Invitrogen no. P36935). Images were taken using a Nikon A1R confocal microscope.

### Statistical analysis.

Statistical analysis was performed using Prism 8 software (GraphPad, Inc.). In experiments containing two groups, statistical analysis was performed using unpaired *t* tests. In experiments with more than two groups, statistical analysis was performed using unpaired one-way analysis of variance (ANOVA), followed by Tukey’s or Bonferroni’s *post hoc* analysis as appropriate. *P* values of <0.05 were considered significant.

### Data availability.

The RNA-seq data set generated in this publication has been deposited in the NCBI database under BioProject accession number GSE162393.

## Supplementary Material

Supplemental file 1

Supplemental file 2
