# Supplementary material for: Elevated Extracellular cGMP Produced after Exposure to Enterotoxigenic Escherichia coli Heat-Stable Toxin Induces Epithelial IL-33 Release and Alters Intestinal Immunity
Source: Infect Immun. 2021 Mar 17;89(4):e00707-20. doi: 10.1128/IAI.00707-20 (PMC8090939; doi:10.1128/IAI.00707-20)
Supplement: Supplemental file 1 [file IAI.00707-20-s0001.pdf]

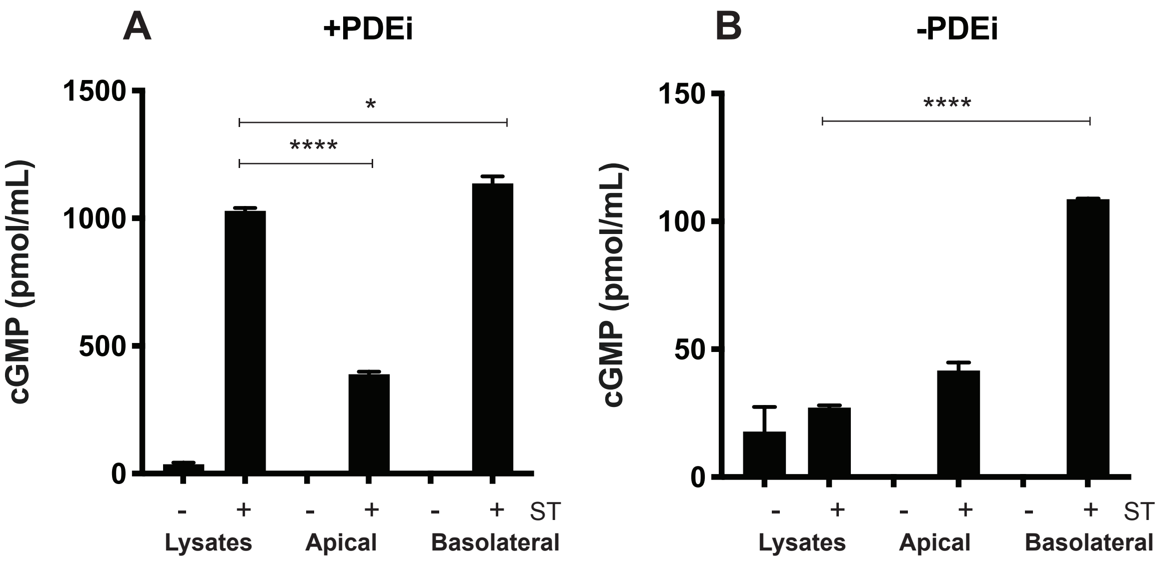

Supplemental Figure 1. cGMP is secreted onto both the apical and basolateral surface of polarized T84 monolayers. ST (200 ng) was applied to T84 monolayers on Transwells for 24-hours in the presence (A) or absence (B) of PDEis. Apical and basolateral secretions were collected and cell lysates were prepared. As shown, in the presence of PDEis, 24-hour ST intoxication induces cGMP localization into both the apical and basolateral compartments, with a preference for basolateral secretion (A). In the absence of PDEis, 24-hour ST intoxication induces much less cGMP localization, but basolateral cGMP accumulation remains significant (B).

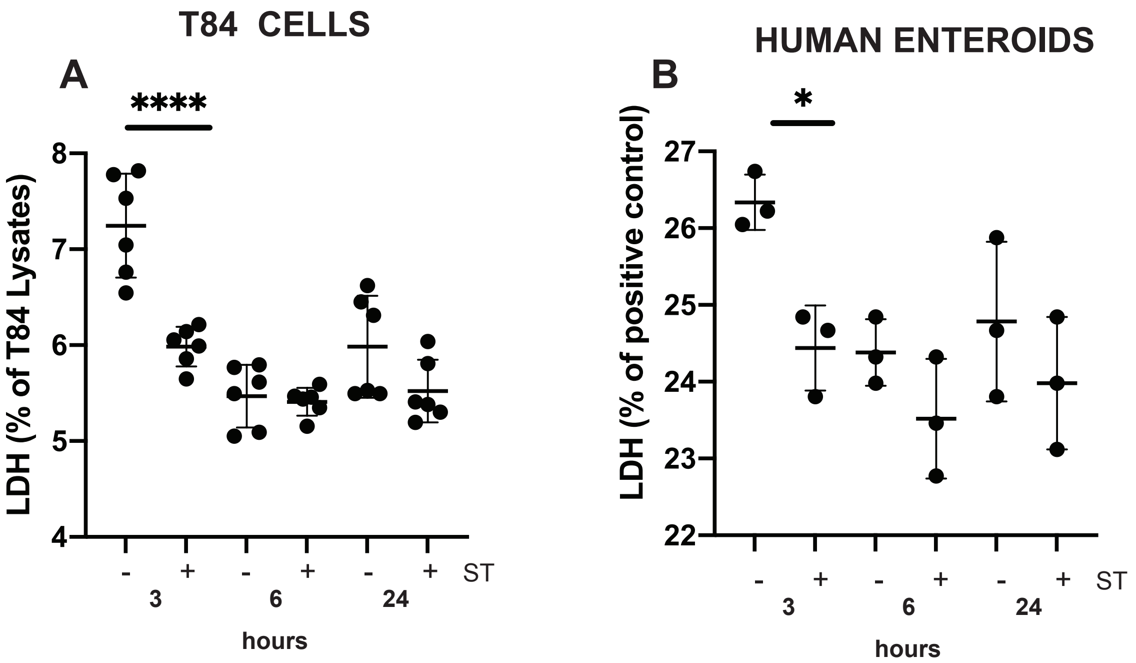

Supplemental Figure 2. ST (100 ng) was applied to T84 cells (A) or human enteroid monolayers (B) for 3-, 6-, or 24-hours. Following intoxication, aliquots were collected from the supernatants and assessed for cell death using the lactate dehydrogenase (LDH) assay. As shown, ST intoxication does not significantly increase the amount of LDH measured in supernatants following 3-, 6-, or 24-hours. This data shows that ST does not induce epithelial cell apoptosis. Postitive control for B is from the Pierce LDH cytotoxicity kit (Thermo 88953).

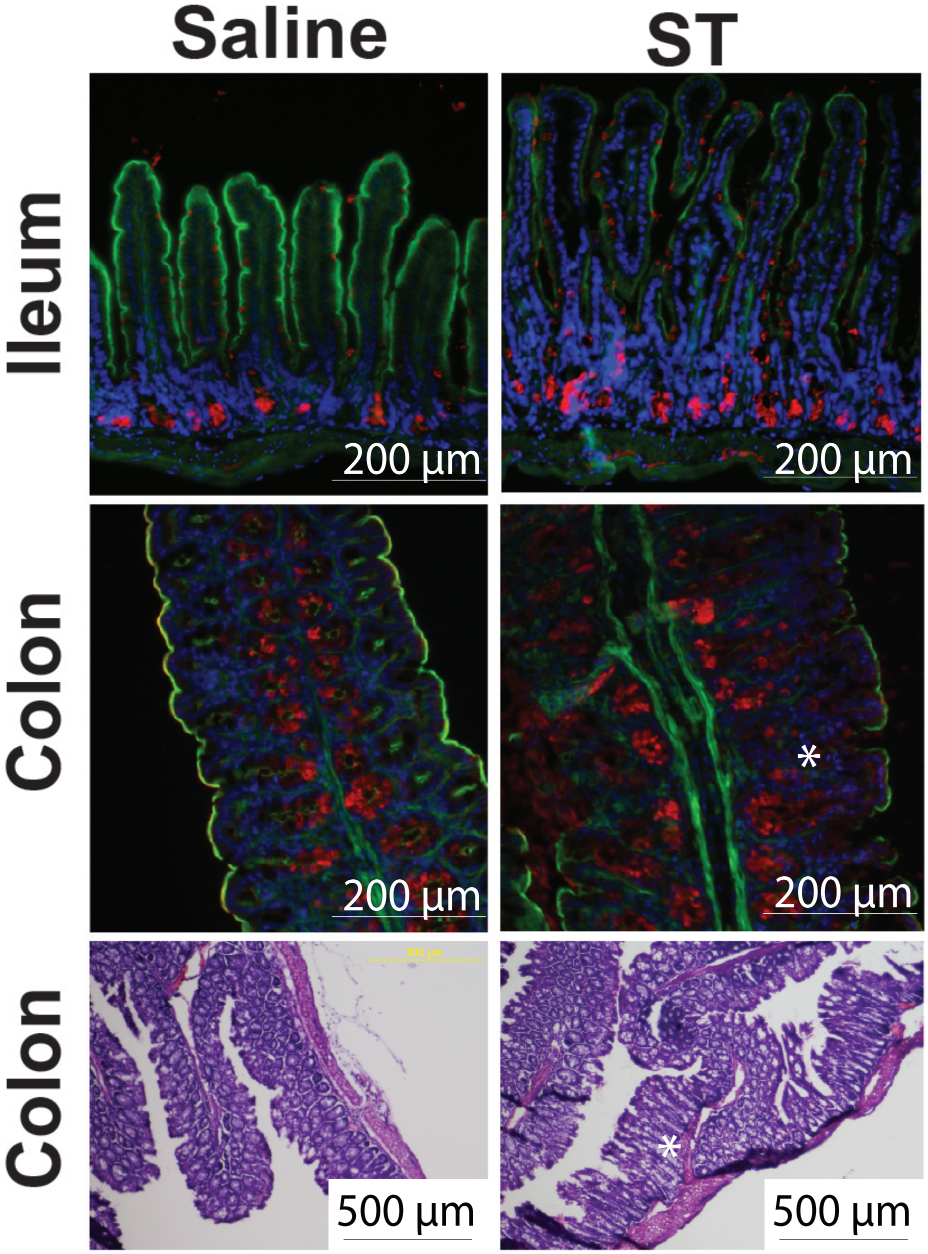

Supplemental Figure 3. Microscopy of representative illeal and colonic tissue from animals treated with saline or ST. Immunofluorescence panel stains include Phalloidin (green), DAPI (blue), and WGA (red). Stars indicate areas of interest. F-actin staining is more diffuse in ST treated ileal tissue and vili are longer (and more distended) in ST-treated ileal tissue. Crypt architecture is intact in saline treated colonic tissue, but stretched in ST treated colonic tissue. Standard H&E stain of colonic tissue. Unless indicated otherwise, values are means + standard deviations.

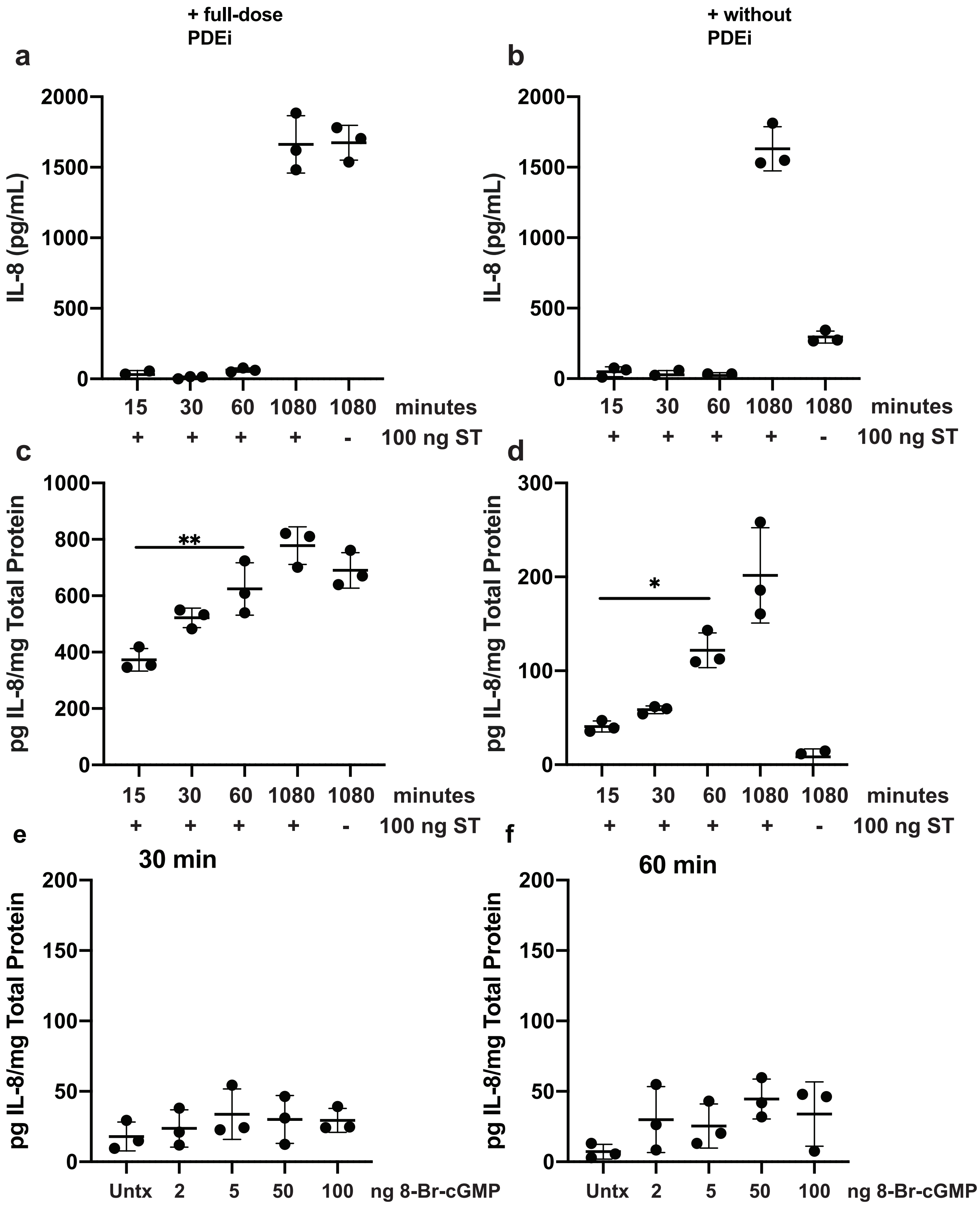

Supplemental Figure 4. ST induction of IL-8 is independent of cGMP. T84 cells were pre-treated with (A, C) and without (B, D) full-dose PDEis and treated kinetically with 100 ng ST. IL-8 levels were determined in both T84 secretions (A, B) and lysates (C,D). In T84 cells treated with full-dose PDEis, IL-8 is found in T84 secretions in both treated and untreated groups at 1080 min after ST intoxication (A). In T84 cells that did not undergo PDEi pre-treatment, IL-8 is found only in secretions in treated cells after 1080 min ST intoxication (B). IL-8 is significantly induced in T84 cell lysates after 60 min ST treatment in cells with PDEi pre-treatment (C) and cells that were not treated with PDEis (D). Cell-permeable cGMP analog 8-Br-cGMP was applied to T84 cells for 30- (E) or 60- (F) minutes at increasing doses and IL-8 levels were determined.

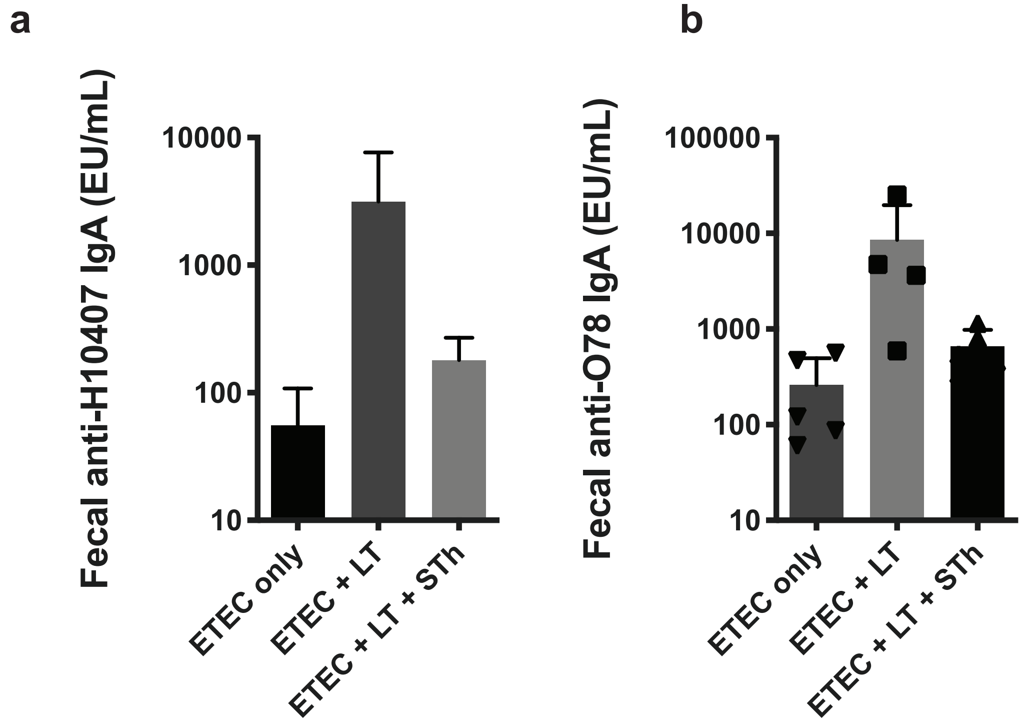

Supplemental Figure 5. ST decreases LT-induced antibody production against ETEC antigens. 6-8 week old BALB/c females (n=5 per group) were immunized orally with 5e9 CFU of heat-killed ETEC H10407 and 10 µg LT with and without 25 µg of ST. The presence of ST in the immunization scheme decreased the amount of fecal anti-H10407 IgA (A) and fecal anti-O78 (B) that is otherwise induced by LT alone.

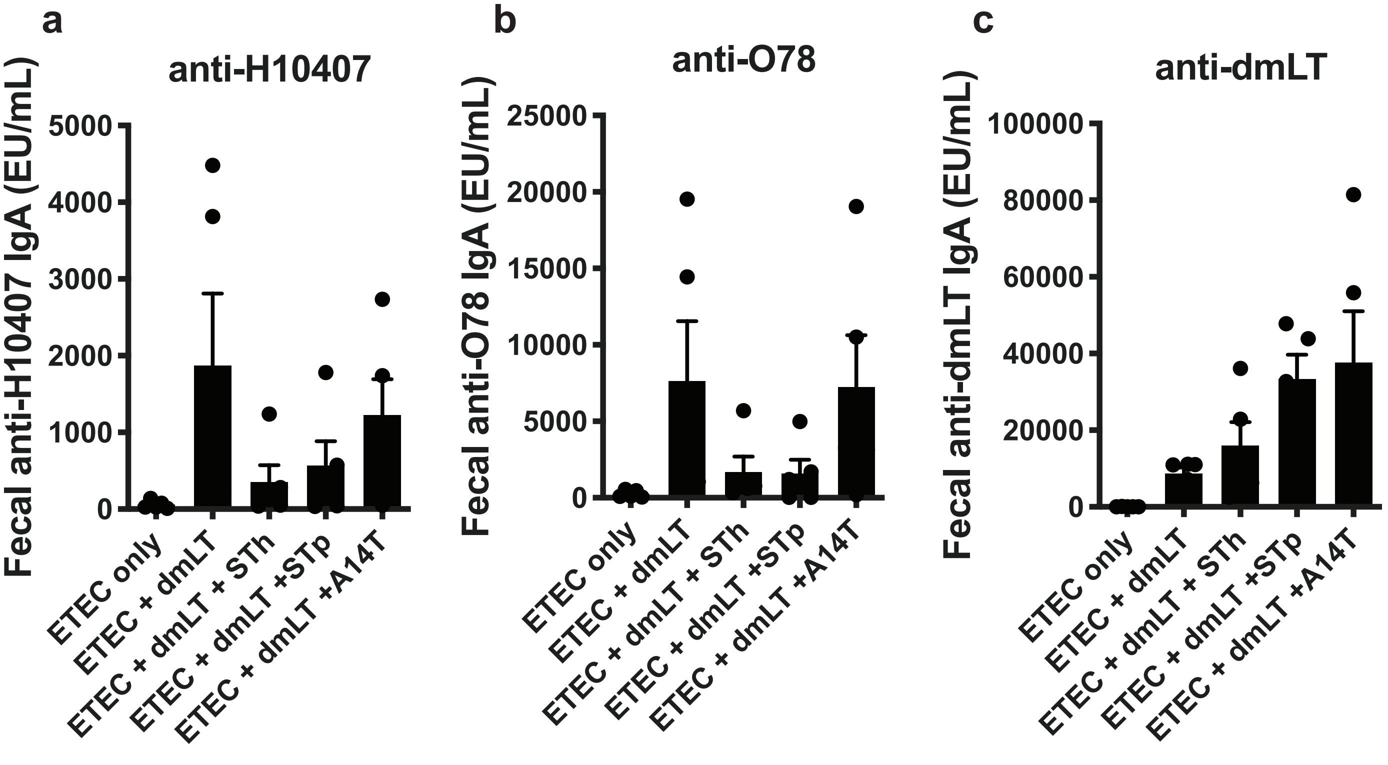

Supplemental Figure 6. Inactive ST toxoid A14T does not suppress IgA against ETEC antigens. 6-8 week old BALB/c females (n=5 per group) were immunized orally with 5e9 CFU of heat-killed ETEC H10407 and 25 µg dmLT with and without 25 µg of STp, STh, or detoxified ST mutant A14T. Fecal anti-H10407 IgA (A) and anti-O78 IgA (B) are lessened in the presence of both STp and STh variants, while detoxified ST A14T shows no dampening of mucosal IgA production. Fecal anti-dm-LT IgA (C) is not lessened by STp, STh, or ST A14T.
